# 2-[2-(2-Anilino-4-oxo-3,4-dihydro­quinazolin-3-yl)phen­oxy]-3-phenyl­quinazolin-4(3*H*)-one methanol hemisolvate

**DOI:** 10.1107/S1600536810028011

**Published:** 2010-07-21

**Authors:** Tao Gao, Xiao-Bao Chen, Xu-Hong Yang, Xiang Wang

**Affiliations:** a Faculty of Chemistry and Life Science, Xianning University, Xianning 437100, Hubei, People’s Republic of China; b Institute of Medicinal Chemistry, Hubei Medical University, Shiyan 442000, Hubei, People’s Republic of China; c College of Life and Environmental Science, Kaili University, Kaili 556000, Guizhou, People’s Republic of China

## Abstract

In the title compound, C_34_H_23_N_5_O_3_·0.5CH_3_OH, each pyrimid­in­one heterocycle and its adjacent benzene ring are almost coplanar, making dihedral angles of 0.69 (13) and 1.87 (13)°. The lower pyrimidinone ring makes a dihedral angle of 40.41 (15)° with the —NH— bonded phenyl ring. O—H⋯O hydrogen bonds and weak C—H⋯π inter­actions are observed in the crystal structure. The methanol solvent molecule is disordered over two positions of equal occupancy.

## Related literature

For the biological activity of quinazoline-4(3*H*)-one derivatives, see: Pandeya *et al.*(1999 ▶); Shiba *et al.* (1997 ▶); Malamas & Millen (1991 ▶); Mannschreck *et al.* (1984 ▶); Kung *et al.* (1999 ▶); Bartroli *et al.* (1998 ▶); Palmer *et al.* (1997 ▶); Tsou *et al.* (2001 ▶); Matsuno *et al.*(2002 ▶). For the synthesis of the title compound, see: Yang *et al.* (2008 ▶).
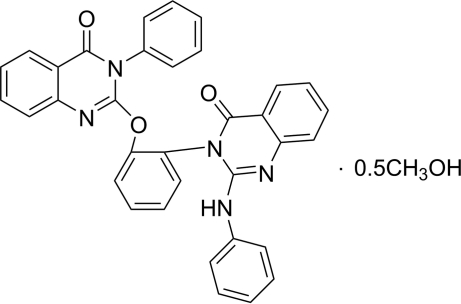


## Experimental

### 

#### Crystal data

C_34_H_23_N_5_O_3_·0.5CH_4_O
*M* *_r_* = 565.60
Monoclinic, 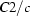

*a* = 24.268 (2) Å
*b* = 16.5049 (14) Å
*c* = 15.2929 (12) Å
β = 110.382 (1)°
*V* = 5741.9 (8) Å^3^
*Z* = 8
Mo *K*α radiation
μ = 0.09 mm^−1^
*T* = 292 K
0.32 × 0.25 × 0.24 mm

#### Data collection

Bruker SMART APEX CCD area-detector diffractometer
Absorption correction: multi-scan (*SADABS*; Sheldrick, 2001 ▶) *T*_min_ = 0.963, *T*_max_ = 0.979
16455 measured reflections
5631 independent reflections
3943 reflections with *I* > 2σ(*I*)
*R*_int_ = 0.022

#### Refinement

*R*[*F* ^2^ > 2σ(*F* ^2^)] = 0.062
*wR*(*F* ^2^) = 0.228
*S* = 1.04
5631 reflections
397 parameters
12 restraints
H-atom parameters constrained
Δρ_max_ = 0.78 e Å^−3^
Δρ_min_ = −0.26 e Å^−3^

### 

Data collection: *SMART* (Bruker, 2000 ▶); cell refinement: *SAINT* (Bruker, 2000 ▶); data reduction: *SAINT*; program(s) used to solve structure: *SHELXS97* (Sheldrick, 2008 ▶); program(s) used to refine structure: *SHELXL97* (Sheldrick, 2008 ▶); molecular graphics: *SHELXTL* (Sheldrick, 2008 ▶); software used to prepare material for publication: *SHELXTL*.

## Supplementary Material

Crystal structure: contains datablocks global, I. DOI: 10.1107/S1600536810028011/bq2224sup1.cif

Structure factors: contains datablocks I. DOI: 10.1107/S1600536810028011/bq2224Isup2.hkl

Additional supplementary materials:  crystallographic information; 3D view; checkCIF report

## Figures and Tables

**Table 1:** Hydrogen-bond geometry (Å, °) *Cg*1 is the centroid of the C29–C34 ring.

*D*—H⋯*A*	*D*—H	H⋯*A*	*D*⋯*A*	*D*—H⋯*A*
O5—H35⋯O5^i^	0.96	0.96	1.711 (7)	125
C2—H2⋯*Cg*1^ii^	0.93	2.75	3.617 (3)	155

Symmetry codes: (i) 
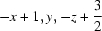
; (ii) 
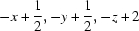
.

**Fig. 1.**
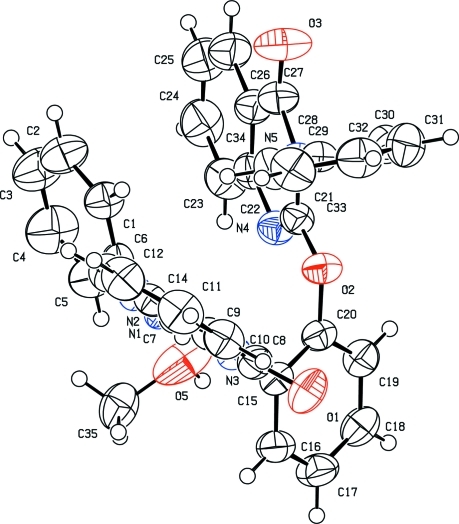
View of the molecular structure of (I), showing the atom labelling schemeand with displacement ellipsoids drawn at the 50% probability level.

**Fig. 2.**
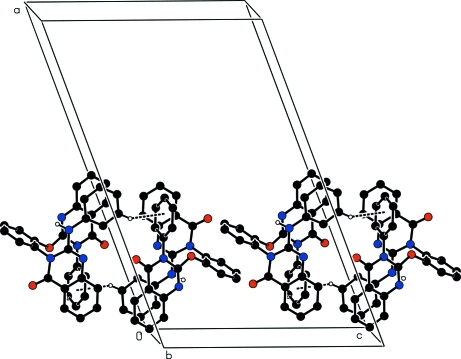
A partial view of the crystal packing of (I). Dashed lines show C-H···π interaction.

## supplementary crystallographic information

### Comment

Quinazoline-4(3*H*)-one derivatives have numerous biological properties. Some of these activities include antimicrobial (Pandeya *et al.*, 1999 and Shiba *et al.*, 1997), antidiabetic (Malamas & Millen, 1991), anticonvulsant (Mannschreck *et al.*, 1984), antibacterial (Kung *et al.*, 1999), antifungal (Bartroli *et al.*, 1998), protein tyrosine kinase inhibitors (Palmer *et al.*, 1997), EGFR inhibitors (Tsou *et al.*,2001) and PDGFR phosphorylation inhibitors (Matsuno *et al.*, 2002). We have recently focused on the synthesis of heterocyclic compounds using an aza-Wittig reaction. We present here the crystal structure of the title compound, (I) (Fig. 1), which can be used as a precursor for obtaining bioactive molecules.

In the crystal structure, the pyrimidinone heterocycle and the adjacent benzene ring are almost planar and inclined at 0.69 (13) ° and 1.87 (13) °. The crystal structure (Fig. 2) is stabilized by weak intermolecular C—H···π hydrogen bonds (Table 1).

### Experimental

To a solution of iminophosphorane (1.40 g, 3.0 mmol) in anhydrous THF (10 ml) was added isocyanatobenzene (3 mmol) under nitrogen at room temperature. After reaction, the mixture was allowed to stand for 10 h at 273–278 K, the solvent was removed under reduced pressure and diethyl ether/petroleum ether (1:2 *v*/*v*, 20 ml) was added to precipitate triphenylphosphine oxide. After filtration, the solvent was removed to give 1-phenyl- 3-(2-ethoxycarbonylphenyl) carbodiimide, which was used directly without further purification. To a solution of 1-phenyl- 3-(2-ethoxycarbonylphenyl) carbodiimide in THF (15 ml) was added 2-aminophenol (1.5 mmol). After the reaction mixture was allowed to stand for 0.5 h, the solvent was removed and anhydrous ethanol (10 ml) with several drops of EtONa in EtOH was added. The mixture was stirred for 2 h at room temperature. The solution was concentrated under reduced pressure and the residue was recrystallized from ethanol to give the title compound. The product was recrystallized from methanol-dichloromethane (1:2 *v*/*v*, 20 ml) at room temperature to give crystals suitable for X-ray diffraction (yield 74%).

### Refinement

All the carbon-bonded hydrogen atoms were located at theire ideal positons with C—H=0.93Å (aromatic) and 0.96Å (methyl), and *U*_iso_(H) = 1.2*U*_eq_C for aromatic and 1.5 *U*_eq_C for methyl hydrogen atoms, respectively. H atoms bonded to N and O atoms were found from the difference maps and then refined with distance restraints of N—H=0.91 (2)Å and O—H=0.96 (2) Å. The thermal factors were set k times of their carriar atoms (k =1.2 for N and 1.5 for O atoms). H20 and H35 was set attached to N1 and O5 atoms, respectively, and they were both constrained to be at their ideal positions.

### Crystal data

**Table:**

C_34_H_23_N_5_O_3_·0.5CH_4_O	*F*(000) = 2360
*M**_r_* = 565.60	*D*_x_ = 1.309 Mg m^−^^3^
Monoclinic, *C*2/*c*	Mo *K*α radiation, λ = 0.71073 Å
Hall symbol: -C 2yc	Cell parameters from 3884 reflections
*a* = 24.268 (2) Å	θ = 2.5–23.6°
*b* = 16.5049 (14) Å	µ = 0.09 mm^−^^1^
*c* = 15.2929 (12) Å	*T* = 292 K
β = 110.382 (1)°	Block, colorless
*V* = 5741.9 (8) Å^3^	0.32 × 0.25 × 0.24 mm
*Z* = 8	

### Data collection

**Table:**

Bruker SMART APEX CCD area-detector diffractometer	5631 independent reflections
Radiation source: fine-focus sealed tube	3943 reflections with *I* > 2σ(*I*)
graphite	*R*_int_ = 0.022
φ and ω scans	θ_max_ = 26.0°, θ_min_ = 2.3°
Absorption correction: multi-scan (*SADABS*; Sheldrick, 2001)	*h* = −29→23
*T*_min_ = 0.963, *T*_max_ = 0.979	*k* = −20→20
16455 measured reflections	*l* = −18→18

### Refinement

**Table:**

Refinement on *F*^2^	Primary atom site location: structure-invariant direct methods
Least-squares matrix: full	Secondary atom site location: difference Fourier map
*R*[*F*^2^ > 2σ(*F*^2^)] = 0.062	Hydrogen site location: inferred from neighbouring sites
*wR*(*F*^2^) = 0.228	H-atom parameters constrained
*S* = 1.04	*w* = 1/[σ^2^(*F*_o_^2^) + (0.1434*P*)^2^ + 2.1314*P*] where *P* = (*F*_o_^2^ + 2*F*_c_^2^)/3
5631 reflections	(Δ/σ)_max_ = 0.002
397 parameters	Δρ_max_ = 0.78 e Å^−^^3^
12 restraints	Δρ_min_ = −0.26 e Å^−^^3^

### Special details

**Table:**

Geometry. All e.s.d.'s (except the e.s.d. in the dihedral angle between two l.s. planes) are estimated using the full covariance matrix. The cell e.s.d.'s are taken into account individually in the estimation of e.s.d.'s in distances, angles and torsion angles; correlations between e.s.d.'s in cell parameters are only used when they are defined by crystal symmetry. An approximate (isotropic) treatment of cell e.s.d.'s is used for estimating e.s.d.'s involving l.s. planes.
Refinement. Refinement of *F*^2^ against ALL reflections. The weighted *R*-factor *wR* and goodness of fit *S* are based on *F*^2^, conventional *R*-factors *R* are based on *F*, with *F* set to zero for negative *F*^2^. The threshold expression of *F*^2^ > σ(*F*^2^) is used only for calculating *R*-factors(gt) *etc*. and is not relevant to the choice of reflections for refinement. *R*-factors based on *F*^2^ are statistically about twice as large as those based on *F*, and *R*- factors based on ALL data will be even larger.

### Fractional atomic coordinates and isotropic or equivalent isotropic displacement parameters (Å^2^)

**Table:**

	*x*	*y*	*z*	*U*_iso_*/*U*_eq_	Occ. (<1)
C1	0.33889 (11)	0.39224 (16)	0.95966 (18)	0.0574 (6)	
H1	0.2986	0.3843	0.9429	0.069*	
C2	0.37488 (14)	0.3822 (2)	1.0508 (2)	0.0749 (8)	
H2	0.3587	0.3681	1.0956	0.090*	
C3	0.43447 (15)	0.3929 (3)	1.0766 (2)	0.0890 (10)	
H3	0.4586	0.3857	1.1384	0.107*	
C4	0.45792 (13)	0.4139 (3)	1.0111 (2)	0.0921 (11)	
H4	0.4983	0.4211	1.0284	0.110*	
C5	0.42214 (12)	0.4248 (2)	0.9184 (2)	0.0709 (8)	
H5	0.4385	0.4392	0.8739	0.085*	
C6	0.36227 (10)	0.41392 (14)	0.89331 (17)	0.0478 (5)	
C7	0.27029 (10)	0.43844 (13)	0.76026 (15)	0.0444 (5)	
C8	0.18242 (10)	0.43060 (15)	0.62005 (16)	0.0491 (6)	
C9	0.15259 (10)	0.46910 (14)	0.67699 (16)	0.0477 (5)	
C10	0.09255 (11)	0.48514 (16)	0.6402 (2)	0.0600 (7)	
H10	0.0712	0.4718	0.5786	0.072*	
C11	0.06483 (12)	0.52059 (18)	0.6949 (2)	0.0688 (8)	
H11	0.0247	0.5314	0.6704	0.083*	
C12	0.09658 (13)	0.54019 (18)	0.7862 (2)	0.0689 (8)	
H12	0.0775	0.5642	0.8229	0.083*	
C13	0.15578 (12)	0.52491 (15)	0.8240 (2)	0.0587 (6)	
H13	0.1766	0.5388	0.8857	0.070*	
C14	0.18475 (10)	0.48859 (13)	0.76995 (16)	0.0458 (5)	
C15	0.27546 (10)	0.37942 (14)	0.61502 (15)	0.0458 (5)	
C16	0.29309 (12)	0.42318 (16)	0.55277 (17)	0.0565 (6)	
H16	0.2841	0.4780	0.5433	0.068*	
C17	0.32434 (13)	0.38468 (18)	0.50451 (19)	0.0642 (7)	
H17	0.3371	0.4140	0.4632	0.077*	
C18	0.33669 (12)	0.30324 (18)	0.51737 (18)	0.0614 (7)	
H18	0.3570	0.2777	0.4836	0.074*	
C19	0.31914 (11)	0.25893 (16)	0.58009 (17)	0.0535 (6)	
H19	0.3279	0.2040	0.5893	0.064*	
C20	0.28861 (10)	0.29777 (14)	0.62827 (15)	0.0457 (5)	
C21	0.30048 (10)	0.22726 (13)	0.76894 (16)	0.0457 (5)	
C22	0.38728 (11)	0.19895 (14)	0.88305 (17)	0.0527 (6)	
C23	0.44826 (12)	0.20351 (18)	0.9158 (2)	0.0678 (7)	
H23	0.4675	0.2278	0.8796	0.081*	
C24	0.48029 (14)	0.1718 (2)	1.0022 (2)	0.0782 (9)	
H24	0.5211	0.1757	1.0244	0.094*	
C25	0.45238 (15)	0.1344 (2)	1.0559 (2)	0.0796 (9)	
H25	0.4743	0.1129	1.1138	0.096*	
C26	0.39234 (14)	0.12903 (18)	1.0239 (2)	0.0698 (8)	
H26	0.3735	0.1033	1.0598	0.084*	
C27	0.35942 (12)	0.16190 (15)	0.93765 (17)	0.0547 (6)	
C28	0.29550 (12)	0.15817 (16)	0.90513 (18)	0.0570 (6)	
C29	0.20409 (10)	0.20150 (14)	0.78235 (16)	0.0465 (5)	
C30	0.17127 (12)	0.13978 (17)	0.72933 (18)	0.0598 (7)	
H30	0.1896	0.0934	0.7182	0.072*	
C31	0.11087 (13)	0.1470 (2)	0.6926 (2)	0.0734 (8)	
H31	0.0884	0.1054	0.6564	0.088*	
C32	0.08382 (12)	0.2155 (2)	0.7093 (2)	0.0707 (8)	
H32	0.0431	0.2203	0.6843	0.085*	
C33	0.11721 (13)	0.27667 (19)	0.7630 (2)	0.0713 (8)	
H33	0.0990	0.3230	0.7744	0.086*	
C34	0.17759 (12)	0.26988 (16)	0.8003 (2)	0.0615 (7)	
H34	0.2001	0.3111	0.8372	0.074*	
N1	0.32858 (9)	0.42035 (13)	0.79688 (14)	0.0520 (5)	
N2	0.24407 (8)	0.47305 (12)	0.81070 (13)	0.0486 (5)	
N3	0.24277 (8)	0.41832 (11)	0.66622 (13)	0.0461 (5)	
N4	0.35614 (9)	0.23084 (12)	0.79476 (14)	0.0510 (5)	
N5	0.26758 (9)	0.19548 (12)	0.81838 (13)	0.0494 (5)	
O1	0.15912 (8)	0.40913 (13)	0.53964 (13)	0.0688 (5)	
O2	0.26452 (7)	0.25565 (10)	0.68598 (11)	0.0513 (4)	
O3	0.26588 (9)	0.12670 (15)	0.94629 (15)	0.0855 (7)	
H20	0.3454	0.4038	0.7553	0.128*	
C35	0.5532 (2)	0.4402 (4)	0.8065 (4)	0.0697 (15)	0.50
H35A	0.5858	0.4492	0.7857	0.105*	0.50
H35B	0.5644	0.4548	0.8711	0.105*	0.50
H35C	0.5205	0.4729	0.7703	0.105*	0.50
O5	0.53385 (15)	0.3409 (4)	0.7931 (3)	0.0959 (18)	0.50
H35	0.5000	0.3679	0.7500	0.144*	

### Atomic displacement parameters (Å^2^)

**Table:**

	*U*^11^	*U*^22^	*U*^33^	*U*^12^	*U*^13^	*U*^23^
C1	0.0490 (14)	0.0654 (16)	0.0556 (15)	0.0017 (12)	0.0153 (12)	0.0043 (12)
C2	0.0726 (19)	0.090 (2)	0.0585 (17)	0.0144 (16)	0.0187 (15)	0.0178 (15)
C3	0.071 (2)	0.119 (3)	0.0589 (18)	0.0104 (19)	0.0004 (16)	0.0188 (18)
C4	0.0460 (16)	0.128 (3)	0.081 (2)	−0.0047 (18)	−0.0044 (15)	0.015 (2)
C5	0.0473 (15)	0.094 (2)	0.0680 (17)	0.0001 (14)	0.0156 (13)	0.0115 (16)
C6	0.0442 (12)	0.0444 (12)	0.0483 (13)	0.0035 (10)	0.0080 (10)	−0.0035 (10)
C7	0.0442 (12)	0.0446 (12)	0.0427 (12)	−0.0025 (10)	0.0129 (10)	−0.0002 (9)
C8	0.0472 (13)	0.0523 (13)	0.0431 (13)	0.0004 (10)	0.0100 (10)	0.0033 (10)
C9	0.0455 (12)	0.0461 (12)	0.0508 (13)	−0.0005 (10)	0.0158 (10)	0.0040 (10)
C10	0.0455 (13)	0.0617 (15)	0.0686 (17)	−0.0002 (11)	0.0146 (12)	0.0048 (13)
C11	0.0464 (14)	0.0698 (18)	0.093 (2)	0.0073 (13)	0.0277 (15)	0.0050 (16)
C12	0.0618 (17)	0.0650 (17)	0.091 (2)	0.0062 (14)	0.0415 (17)	−0.0021 (15)
C13	0.0620 (16)	0.0545 (14)	0.0664 (16)	0.0029 (12)	0.0310 (13)	−0.0028 (12)
C14	0.0474 (12)	0.0390 (11)	0.0528 (13)	0.0007 (9)	0.0198 (11)	0.0029 (10)
C15	0.0465 (12)	0.0510 (13)	0.0396 (12)	0.0012 (10)	0.0145 (10)	−0.0007 (10)
C16	0.0686 (16)	0.0552 (14)	0.0501 (14)	0.0009 (12)	0.0263 (12)	0.0080 (11)
C17	0.0759 (18)	0.0719 (18)	0.0549 (15)	0.0002 (14)	0.0354 (14)	0.0089 (13)
C18	0.0617 (16)	0.0774 (18)	0.0519 (14)	0.0054 (13)	0.0284 (12)	−0.0057 (13)
C19	0.0525 (14)	0.0556 (14)	0.0519 (13)	0.0061 (11)	0.0174 (11)	−0.0013 (11)
C20	0.0439 (12)	0.0536 (13)	0.0389 (11)	−0.0010 (10)	0.0133 (10)	0.0047 (10)
C21	0.0489 (13)	0.0415 (11)	0.0468 (13)	0.0020 (10)	0.0170 (10)	0.0027 (10)
C22	0.0532 (14)	0.0446 (12)	0.0536 (14)	0.0046 (11)	0.0104 (11)	0.0031 (11)
C23	0.0557 (16)	0.0647 (16)	0.0731 (18)	−0.0019 (13)	0.0101 (14)	0.0070 (14)
C24	0.0592 (17)	0.077 (2)	0.078 (2)	0.0017 (15)	−0.0017 (15)	0.0021 (16)
C25	0.077 (2)	0.078 (2)	0.0643 (18)	0.0122 (16)	−0.0001 (16)	0.0130 (16)
C26	0.078 (2)	0.0664 (17)	0.0570 (16)	0.0106 (15)	0.0134 (14)	0.0137 (14)
C27	0.0611 (15)	0.0498 (13)	0.0492 (14)	0.0069 (11)	0.0143 (12)	0.0060 (11)
C28	0.0609 (15)	0.0572 (15)	0.0525 (14)	0.0055 (12)	0.0191 (12)	0.0130 (12)
C29	0.0480 (13)	0.0471 (12)	0.0458 (12)	−0.0003 (10)	0.0181 (10)	0.0076 (10)
C30	0.0643 (16)	0.0556 (14)	0.0570 (15)	0.0019 (12)	0.0182 (13)	−0.0006 (12)
C31	0.0647 (18)	0.079 (2)	0.0650 (18)	−0.0138 (15)	0.0076 (14)	−0.0048 (15)
C32	0.0490 (15)	0.088 (2)	0.0686 (18)	0.0013 (14)	0.0126 (13)	0.0145 (16)
C33	0.0608 (17)	0.0680 (17)	0.088 (2)	0.0112 (14)	0.0292 (16)	0.0046 (16)
C34	0.0552 (15)	0.0544 (15)	0.0740 (17)	−0.0031 (12)	0.0214 (13)	−0.0056 (13)
N1	0.0453 (11)	0.0629 (12)	0.0463 (11)	0.0034 (9)	0.0140 (9)	−0.0051 (9)
N2	0.0471 (11)	0.0502 (11)	0.0477 (11)	0.0012 (8)	0.0155 (9)	−0.0053 (9)
N3	0.0470 (11)	0.0496 (11)	0.0419 (10)	0.0021 (8)	0.0158 (8)	−0.0001 (8)
N4	0.0492 (11)	0.0518 (11)	0.0505 (11)	0.0022 (9)	0.0153 (9)	0.0058 (9)
N5	0.0513 (11)	0.0480 (10)	0.0500 (11)	0.0031 (9)	0.0190 (9)	0.0110 (9)
O1	0.0585 (11)	0.0912 (14)	0.0487 (11)	0.0062 (10)	0.0088 (9)	−0.0088 (10)
O2	0.0474 (9)	0.0591 (10)	0.0468 (9)	0.0021 (7)	0.0156 (7)	0.0142 (7)
O3	0.0739 (14)	0.1109 (17)	0.0767 (14)	0.0072 (12)	0.0324 (11)	0.0445 (13)
C35	0.053 (3)	0.090 (4)	0.062 (3)	−0.010 (3)	0.014 (3)	−0.016 (3)
O5	0.0276 (17)	0.201 (6)	0.058 (2)	−0.005 (2)	0.0139 (16)	0.020 (3)

### Geometric parameters (Å, °)

**Table:**

C1—C6	1.371 (4)	C19—H19	0.9300
C1—C2	1.373 (4)	C20—O2	1.401 (3)
C1—H1	0.9300	C21—N4	1.270 (3)
C2—C3	1.372 (4)	C21—O2	1.349 (3)
C2—H2	0.9300	C21—N5	1.380 (3)
C3—C4	1.357 (5)	C22—C27	1.386 (4)
C3—H3	0.9300	C22—C23	1.389 (4)
C4—C5	1.393 (4)	C22—N4	1.400 (3)
C4—H4	0.9300	C23—C24	1.382 (4)
C5—C6	1.379 (3)	C23—H23	0.9300
C5—H5	0.9300	C24—C25	1.378 (5)
C6—N1	1.419 (3)	C24—H24	0.9300
C7—N2	1.292 (3)	C25—C26	1.369 (4)
C7—N1	1.361 (3)	C25—H25	0.9300
C7—N3	1.398 (3)	C26—C27	1.392 (4)
C8—O1	1.214 (3)	C26—H26	0.9300
C8—N3	1.402 (3)	C27—C28	1.456 (4)
C8—C9	1.458 (3)	C28—O3	1.224 (3)
C9—C10	1.392 (3)	C28—N5	1.404 (3)
C9—C14	1.400 (3)	C29—C30	1.371 (4)
C10—C11	1.373 (4)	C29—C34	1.374 (3)
C10—H10	0.9300	C29—N5	1.448 (3)
C11—C12	1.378 (4)	C30—C31	1.380 (4)
C11—H11	0.9300	C30—H30	0.9300
C12—C13	1.373 (4)	C31—C32	1.375 (4)
C12—H12	0.9300	C31—H31	0.9300
C13—C14	1.394 (3)	C32—C33	1.375 (4)
C13—H13	0.9300	C32—H32	0.9300
C14—N2	1.379 (3)	C33—C34	1.379 (4)
C15—C16	1.376 (3)	C33—H33	0.9300
C15—C20	1.383 (3)	C34—H34	0.9300
C15—N3	1.445 (3)	N1—H20	0.9098
C16—C17	1.383 (4)	C35—O5	1.698 (9)
C16—H16	0.9300	C35—H35A	0.9600
C17—C18	1.376 (4)	C35—H35B	0.9600
C17—H17	0.9300	C35—H35C	0.9600
C18—C19	1.386 (4)	O5—O5^i^	1.711 (7)
C18—H18	0.9300	O5—H35	0.9646
C19—C20	1.372 (3)		
			
C6—C1—C2	120.1 (3)	N4—C21—N5	127.1 (2)
C6—C1—H1	119.9	O2—C21—N5	109.81 (19)
C2—C1—H1	119.9	C27—C22—C23	119.2 (2)
C3—C2—C1	120.6 (3)	C27—C22—N4	122.3 (2)
C3—C2—H2	119.7	C23—C22—N4	118.5 (2)
C1—C2—H2	119.7	C24—C23—C22	119.9 (3)
C4—C3—C2	119.5 (3)	C24—C23—H23	120.1
C4—C3—H3	120.2	C22—C23—H23	120.1
C2—C3—H3	120.2	C25—C24—C23	120.6 (3)
C3—C4—C5	120.7 (3)	C25—C24—H24	119.7
C3—C4—H4	119.6	C23—C24—H24	119.7
C5—C4—H4	119.6	C26—C25—C24	119.9 (3)
C6—C5—C4	119.3 (3)	C26—C25—H25	120.1
C6—C5—H5	120.3	C24—C25—H25	120.1
C4—C5—H5	120.3	C25—C26—C27	120.1 (3)
C1—C6—C5	119.7 (2)	C25—C26—H26	119.9
C1—C6—N1	123.4 (2)	C27—C26—H26	119.9
C5—C6—N1	116.7 (2)	C22—C27—C26	120.2 (3)
N2—C7—N1	120.6 (2)	C22—C27—C28	119.9 (2)
N2—C7—N3	124.1 (2)	C26—C27—C28	119.9 (3)
N1—C7—N3	115.27 (19)	O3—C28—N5	119.7 (2)
O1—C8—N3	120.3 (2)	O3—C28—C27	126.1 (2)
O1—C8—C9	125.4 (2)	N5—C28—C27	114.2 (2)
N3—C8—C9	114.3 (2)	C30—C29—C34	120.8 (2)
C10—C9—C14	120.1 (2)	C30—C29—N5	119.8 (2)
C10—C9—C8	120.6 (2)	C34—C29—N5	119.4 (2)
C14—C9—C8	119.3 (2)	C29—C30—C31	119.5 (3)
C11—C10—C9	120.1 (3)	C29—C30—H30	120.2
C11—C10—H10	120.0	C31—C30—H30	120.2
C9—C10—H10	120.0	C32—C31—C30	120.2 (3)
C10—C11—C12	119.8 (2)	C32—C31—H31	119.9
C10—C11—H11	120.1	C30—C31—H31	119.9
C12—C11—H11	120.1	C31—C32—C33	119.7 (3)
C13—C12—C11	121.2 (3)	C31—C32—H32	120.2
C13—C12—H12	119.4	C33—C32—H32	120.2
C11—C12—H12	119.4	C32—C33—C34	120.5 (3)
C12—C13—C14	120.0 (3)	C32—C33—H33	119.7
C12—C13—H13	120.0	C34—C33—H33	119.7
C14—C13—H13	120.0	C29—C34—C33	119.2 (3)
N2—C14—C13	118.3 (2)	C29—C34—H34	120.4
N2—C14—C9	122.8 (2)	C33—C34—H34	120.4
C13—C14—C9	118.9 (2)	C7—N1—C6	125.7 (2)
C16—C15—C20	120.1 (2)	C7—N1—H20	115.7
C16—C15—N3	120.2 (2)	C6—N1—H20	117.9
C20—C15—N3	119.72 (19)	C7—N2—C14	117.8 (2)
C15—C16—C17	119.2 (2)	C7—N3—C8	121.65 (19)
C15—C16—H16	120.4	C7—N3—C15	120.48 (19)
C17—C16—H16	120.4	C8—N3—C15	117.77 (18)
C18—C17—C16	120.3 (2)	C21—N4—C22	116.2 (2)
C18—C17—H17	119.8	C21—N5—C28	120.2 (2)
C16—C17—H17	119.8	C21—N5—C29	120.49 (18)
C17—C18—C19	120.7 (2)	C28—N5—C29	119.28 (19)
C17—C18—H18	119.7	C21—O2—C20	119.30 (17)
C19—C18—H18	119.7	O5—C35—H35A	109.5
C20—C19—C18	118.6 (2)	O5—C35—H35B	109.5
C20—C19—H19	120.7	H35A—C35—H35B	109.5
C18—C19—H19	120.7	O5—C35—H35C	109.5
C19—C20—C15	121.1 (2)	H35A—C35—H35C	109.5
C19—C20—O2	121.9 (2)	H35B—C35—H35C	109.5
C15—C20—O2	116.73 (19)	C35—O5—O5^i^	104.1 (2)
N4—C21—O2	123.1 (2)		
			
C6—C1—C2—C3	−0.7 (5)	C34—C29—C30—C31	0.9 (4)
C1—C2—C3—C4	0.3 (6)	N5—C29—C30—C31	−177.9 (2)
C2—C3—C4—C5	0.1 (6)	C29—C30—C31—C32	−0.2 (4)
C3—C4—C5—C6	−0.1 (6)	C30—C31—C32—C33	−0.2 (5)
C2—C1—C6—C5	0.7 (4)	C31—C32—C33—C34	0.0 (5)
C2—C1—C6—N1	175.8 (3)	C30—C29—C34—C33	−1.1 (4)
C4—C5—C6—C1	−0.3 (4)	N5—C29—C34—C33	177.7 (2)
C4—C5—C6—N1	−175.8 (3)	C32—C33—C34—C29	0.6 (4)
O1—C8—C9—C10	−0.7 (4)	N2—C7—N1—C6	19.3 (4)
N3—C8—C9—C10	179.9 (2)	N3—C7—N1—C6	−161.8 (2)
O1—C8—C9—C14	178.1 (2)	C1—C6—N1—C7	27.8 (4)
N3—C8—C9—C14	−1.2 (3)	C5—C6—N1—C7	−156.9 (2)
C14—C9—C10—C11	0.4 (4)	N1—C7—N2—C14	−179.0 (2)
C8—C9—C10—C11	179.3 (2)	N3—C7—N2—C14	2.3 (3)
C9—C10—C11—C12	0.0 (4)	C13—C14—N2—C7	179.1 (2)
C10—C11—C12—C13	0.0 (5)	C9—C14—N2—C7	−0.6 (3)
C11—C12—C13—C14	−0.3 (4)	N2—C7—N3—C8	−3.6 (3)
C12—C13—C14—N2	−179.0 (2)	N1—C7—N3—C8	177.6 (2)
C12—C13—C14—C9	0.7 (4)	N2—C7—N3—C15	−179.8 (2)
C10—C9—C14—N2	179.0 (2)	N1—C7—N3—C15	1.3 (3)
C8—C9—C14—N2	0.1 (3)	O1—C8—N3—C7	−176.6 (2)
C10—C9—C14—C13	−0.7 (3)	C9—C8—N3—C7	2.8 (3)
C8—C9—C14—C13	−179.6 (2)	O1—C8—N3—C15	−0.2 (3)
C20—C15—C16—C17	−0.5 (4)	C9—C8—N3—C15	179.16 (19)
N3—C15—C16—C17	−180.0 (2)	C16—C15—N3—C7	−103.9 (3)
C15—C16—C17—C18	1.2 (4)	C20—C15—N3—C7	76.6 (3)
C16—C17—C18—C19	−1.3 (4)	C16—C15—N3—C8	79.7 (3)
C17—C18—C19—C20	0.7 (4)	C20—C15—N3—C8	−99.8 (3)
C18—C19—C20—C15	0.0 (4)	O2—C21—N4—C22	179.8 (2)
C18—C19—C20—O2	173.7 (2)	N5—C21—N4—C22	0.0 (4)
C16—C15—C20—C19	−0.1 (4)	C27—C22—N4—C21	3.1 (3)
N3—C15—C20—C19	179.4 (2)	C23—C22—N4—C21	−178.1 (2)
C16—C15—C20—O2	−174.2 (2)	N4—C21—N5—C28	−3.7 (4)
N3—C15—C20—O2	5.3 (3)	O2—C21—N5—C28	176.5 (2)
C27—C22—C23—C24	−0.5 (4)	N4—C21—N5—C29	175.2 (2)
N4—C22—C23—C24	−179.3 (3)	O2—C21—N5—C29	−4.6 (3)
C22—C23—C24—C25	1.0 (5)	O3—C28—N5—C21	−176.4 (2)
C23—C24—C25—C26	−0.5 (5)	C27—C28—N5—C21	3.9 (3)
C24—C25—C26—C27	−0.6 (5)	O3—C28—N5—C29	4.7 (4)
C23—C22—C27—C26	−0.6 (4)	C27—C28—N5—C29	−175.0 (2)
N4—C22—C27—C26	178.2 (2)	C30—C29—N5—C21	92.6 (3)
C23—C22—C27—C28	178.7 (2)	C34—C29—N5—C21	−86.1 (3)
N4—C22—C27—C28	−2.5 (4)	C30—C29—N5—C28	−88.5 (3)
C25—C26—C27—C22	1.2 (4)	C34—C29—N5—C28	92.8 (3)
C25—C26—C27—C28	−178.1 (3)	N4—C21—O2—C20	−6.0 (3)
C22—C27—C28—O3	179.3 (3)	N5—C21—O2—C20	173.79 (18)
C26—C27—C28—O3	−1.3 (4)	C19—C20—O2—C21	73.6 (3)
C22—C27—C28—N5	−1.0 (3)	C15—C20—O2—C21	−112.4 (2)
C26—C27—C28—N5	178.3 (2)		

Symmetry codes: (i) −*x*+1, *y*, −*z*+3/2.

### Hydrogen-bond geometry (Å, °)

**Table:**

Cg1 is the centroid of the C29–C34 ring.

**Table:**

*D*—H···*A*	*D*—H	H···*A*	*D*···*A*	*D*—H···*A*
O5—H35···O5^i^	0.96	0.96	1.711 (7)	125
C2—H2···Cg1^ii^	0.93	2.75	3.617 (3)	155

Symmetry codes: (i) −*x*+1, *y*, −*z*+3/2; (ii) −*x*+1/2, −*y*+1/2, −*z*+2.
